# Exponential decay of spatial correlation in driven diffusive system: A universal feature of macroscopic homogeneous state

**DOI:** 10.1038/srep19652

**Published:** 2016-01-25

**Authors:** Qing-Yi Hao, Rui Jiang, Mao-Bin Hu, Bin Jia, Wen-Xu Wang

**Affiliations:** 1 School of Mathematics and Computational Science, Anqing Teachers College, Anqing 246133, P. R. China; 2 School of Systems Science, Beijing Normal University, Beijing, 100875, P.R. China; 3 School of Traffic and Transportation, Beijing Jiaotong University, Beijing 100044, P.R. China; 4 School of Engineering Science, University of Science and Technology of China, Hefei 230026, P. R. China; 5 Business School, University of Shanghai for Science and Technology, Shanghai 200093, China

## Abstract

Driven diffusive systems have been a paradigm for modelling many physical, chemical, and biological transport processes. In the systems, spatial correlation plays an important role in the emergence of a variety of nonequilibrium phenomena and exhibits rich features such as pronounced oscillations. However, the lack of analytical results of spatial correlation precludes us from fully understanding the effect of spatial correlation on the dynamics of the system. Here we offer precise analytical predictions of the spatial correlation in a typical driven diffusive system, namely facilitated asymmetric exclusion process. We find theoretically that the correlation between two sites decays exponentially as their distance increases, which is in good agreement with numerical simulations. Furthermore, we find the exponential decay is a universal property of macroscopic homogeneous state in a broad class of 1D driven diffusive systems. Our findings deepen the understanding of many nonequilibrium phenomena resulting from spatial correlation in driven diffusive systems.

Driven diffusive systems are of current interest in nonequilibrium statistical mechanics due to their rich and complex dynamic features^1^,^2^,^3^,^4^,^5^. A simple and typical model in these systems is the asymmetric simple exclusion process (ASEP) describing particles hopping with hard-core repulsion along a one dimensional lattice unidirectionally. The ASEP was introduced in 1968 by MacDonald and Gibbs to model protein synthesis in organisms^6^. Recently, numerous variants of ASEP have been developed to model biological transport^7^,^8^,^9^,^10^, polymer dynamics in dense media^11^, diffusion through membrane channels^12^, traffic flow^13^,^14^, and so on. Despite relatively simple rules, the ASEP and related models show a range of nontrivial macroscopic phenomena such as boundary induced and bulk induced phase transitions^15^,^16^,^17^,^18^, spontaneous symmetry breaking^19^,^20^, phase separation^21^,^22^,^23^,^24^,^25^, and thus serve as basic tools to investigate the systems far from thermal equilibrium^26^,^27^,^28^.

In driven diffusive systems, spatial correlation plays an important role in the formation of the diverse nonequilibrium phenomena^29^,^30^. As an exceptional case, in the basic ASEP, the correlation is absent^2^,^31^. Thus, the simple mean-field analysis is able to offer the exact current 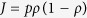, where *ρ* is the system density and *p* is the hopping rate. In contrast, spatial correlation usually exists in general situations, which makes the traditional mean-field analysis incapable of rendering the theoretical solution. In most cases, numerical simulation is still the exclusive tool to explore the spatial and temporal correlation in driven diffusive systems. The increased use of cluster mean-field, is another method of testing the correlations^32^. Some interesting phenomena have been observed from simulations. For instance, Gupta *et al.* found that density correlations display pronounced oscillations in both space and time, as a consequence of particles with extended length. The density autocorrelation has been found to decay exponentially at time increases, except at a special density when it decays as a power law^33^.

Here we aim to offer analytical results of the spatial correlation in a representative driven diffusive system, namely facilitated asymmetric exclusion process that is subject to a generalized class of ASEP models. Specifically, in the model, the hopping probability of a particle depends on the occupancies of two neighboring sites: one ahead and one behind^34^. The model was proposed to study nonequilibrium absorbing state phase transitions^35^. It is relevant to several facts such as the particle mobility decreases as the local density increases in glassy dynamics^36^, and a moving particle can exert a hydrodynamic force that pushes other particles along in molecular motor models^37^. Moreover, due to particle-hole symmetry, the facilitated exclusion process is the counterpart of the ASEP with next-nearest-neighbor interaction as studied in ref. 14.

To explore the spatial correlation analytically, we first derive the joint occupancy probabilities in the facilitated asymmetric exclusion process theoretically. The formula of the joint occupancy probabilities allows us to provide the exact formula of the spatial correlation between any two sites in the model. The analytical results have been validated and are in good agreement with numerical simulations. Furthermore, we explore the spatial correlation in several other driven diffusive systems, finding that the spatial correlation decays exponentially in all the investigated systems. These observations suggest that the exponential decay of spatial correlation is a universal feature in 1D driven diffusive systems with macroscopic homogeneous state. The findings considerably deepen our understanding of the emergence of many nonequilibrium phenomena that stem from the nonlinear spatial correlation, such as the jamming in a variety of transport systems in biology and social systems.

## Results and Discussions

**Figure 1:**
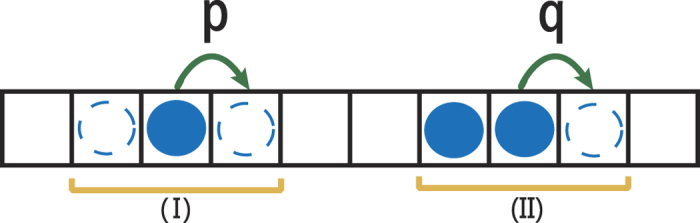
Sketch of the model. A particle moves to the front empty site with probability *p* if the rear site is empty(configuration I). Otherwise, if the rear site is occupied, the particle moves to the front empty site with probability *q* (configuration II). In the case of *p* = *q*, the model reduces to the basic ASEP. The filled circles indicate sites occupied by particles, and the dotted-open circles denote empty sites. The absence of circles means the site is empty or occupied.

The sketch of the facilitated exclusion process studied in this paper is shown in Fig. 1. The model rules are as follows. A particle at site *i* moves to site *i* + 1 with probability *p* if the front site *i* + 1 is empty and the rear site *i* − 1 is also empty. Otherwise, if the rear site *i* − 1 is occupied, the particle at site *i* hops to site *i* + 1 with probability *q* if site *i* + 1 is empty. In the model, random update rules and periodic boundary conditions are employed. In the special case *p* = *q*, the model reduces to the basic ASEP.

We consider the four joint occupancy probabilities *P*(*τ*_*i*_, *τ*_*i*+1_). Here 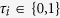 denoting that site *i* is empty (*τ*_*i*_ = 0) or occupied (*τ*_*i*_ = 1). For the convenience of expression, we also use *x*, *y* and *z* to denote *P*(1, 1), *P*(1, 0) and *P*(0, 0) respectively. Note that due to symmetry, one has *P*(1, 0) = *P*(0, 1). Via a two-cluster mean field analysis of the model (see section Methods), we can obtain


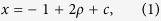



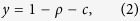



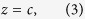


where


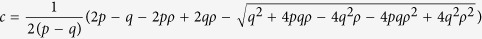


when *p* ≠ *q*. In the special case *p* = *q*, the solution is *x* = *ρ*^2^, *y* = *ρ*(1 − *ρ*), *z* = (1 − *ρ*)^2^. As demonstrated in ref. 38, the two cluster mean field results are exact solution of the system.

Next we investigate the correlations in the system based on the exact solution. We define the correlation between two sites as


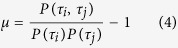


Obviously *μ* = 0 if and only if there is no correlation between sites *i* and *j*. Note that there are four correlation coefficients, and we let *μ*_1_ (*μ*_2_, *μ*_3_, *μ*_4_) denote the one with *τ*_*i*_ = 0 and *τ*_*j*_ = 0 (*τ*_*i*_ = 1 and *τ*_*j*_ = 0, *τ*_*i*_ = 0 and *τ*_*j*_ = 1, *τ*_*i*_ = 1 and *τ*_*j*_ = 1). Thus the correlation coefficient, say *μ*_1_, can be expressed as


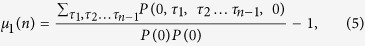


with *n* denoting the distance between sites *i* and *j*. Note that the classical correlation function 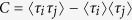 is related to *μ*_4_ via 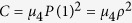.

Using the joint occupancy probabilities, we can derive the correlation coefficients (see section Methods)


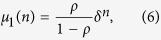



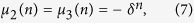


and


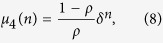


where


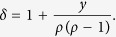


Note that when *y* > *ρ*(1 − *ρ*), the four correlation coefficients vary alternatively between positive and negative values. In the special case *p* = *q*, *μ* = 0 as expected because *y* = *ρ*(1 − *ρ*).

**Figure 2:**
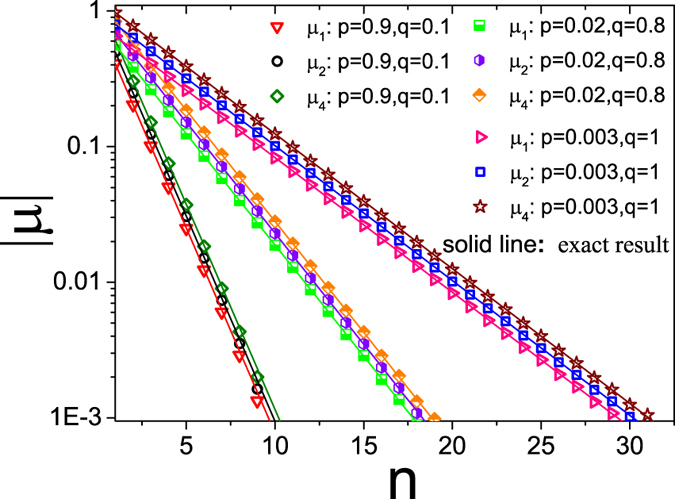
Plot of absolute value of correlation coefficients 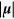 versus distance *n*. Here the density *ρ* = 0.45. The symbols denote the simulation results with system size *L* = 6000, the lines indicate the analysis results from Eqs (6)–(8).

Obviously, in the case of *ρ* = 0.5, 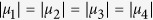. Figure 2 shows the exponential relationship between 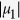, 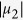, 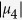 and *n*. The Monte Carlo simulations and the analytical expressions are in perfect agreement.

Now we investigate physical implication of the exponential decay of correlation in the driven diffusive systems. To this end, we study four different models of driven diffusive systems.

- The Katz-Lebowitz-Spohn (KLS) model^39^,^40^. In the KLS model, particle hops with rate as follows: 1100 → 1010 with rate 1 + *ε*, 0101 → 0011 with rate 1 − *ε*, 0100 → 0010 with rate 1 + *δ*, 1101 → 1011 with rate 1 − *δ*. Here “1” denotes a particle and “0” denotes an empty site.
- The Dierl-Maass-Einax (DME) model^41^. In the DME model, particle hops from site *i* to site *i* + 1 with rate 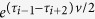.
- The bus route model^42^. In the model, a particle (bus) hops with rate 1 if there is no passenger at the site. Otherwise the particle hops with rate *p* < 1. At each empty site, passengers arrive with rate *λ*.
- The bidirectional two-lane model^25^. In the model, particles move with opposite direction on two parallel lanes and do not change lane. The inter-lane interaction is implemented as particles slow down when there is a particle at the same site in the other lane, which mimics narrow road section. In this case, particle hopping rate *p* < 1. Otherwise, particle hops with rate 1.

**Figure 3:**
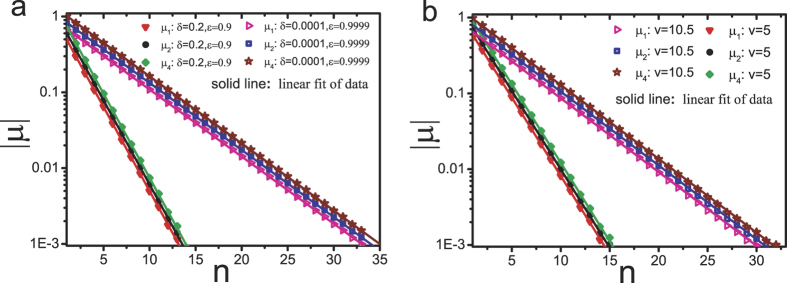
Plot of absolute value of correlation coefficients 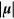 versus distance *n* in (a) the Katz-Lebowitz-Spohn model and (b) the Dierl-Maass-Einax model. Here the density *ρ* = 0.45. The symbols denote the simulation results with system size *L* = 6000, the lines indicate the linear fit.

Although we cannot derive the exact expression of correlation, numerical simulations show that the correlation also decays exponentially in the KLS model and the DME model, see Fig. 3. Note that in the KLS model, the DME model, and the facilitated ASEP, the system is always macroscopically homogeneous.

**Figure 4:**
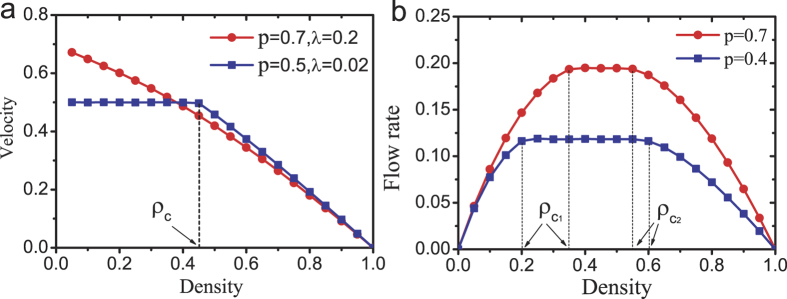
The velocity or flow rate versus system density. (**a**) The bus route model with system size *L* = 6000. Note that when *p* = 0.7, *λ* = 0.2, the critical density *ρ*_*c*_ = 0 and bus bunching disappears. (**b**) The bidirectional two-lane model with system size *L* = 6000.

**Figure 5:**
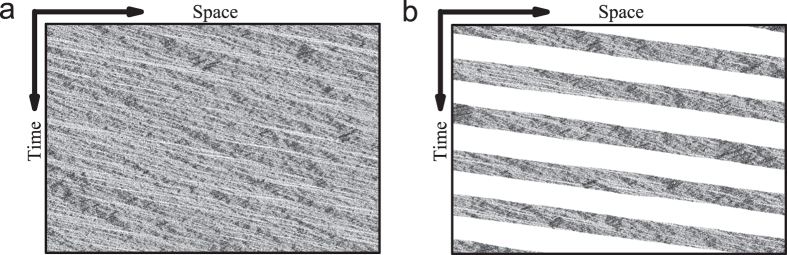
The spatiotemporal patterns in the bus route model. (**a**) Homogeneous state, the density *ρ* = 0.4 and *p* = 0.7, *λ* = 0.2. (**b**) Bus bunching state, the density *ρ* = 0.2 and *p* = 0.5, *λ* = 0.02.

**Figure 6:**
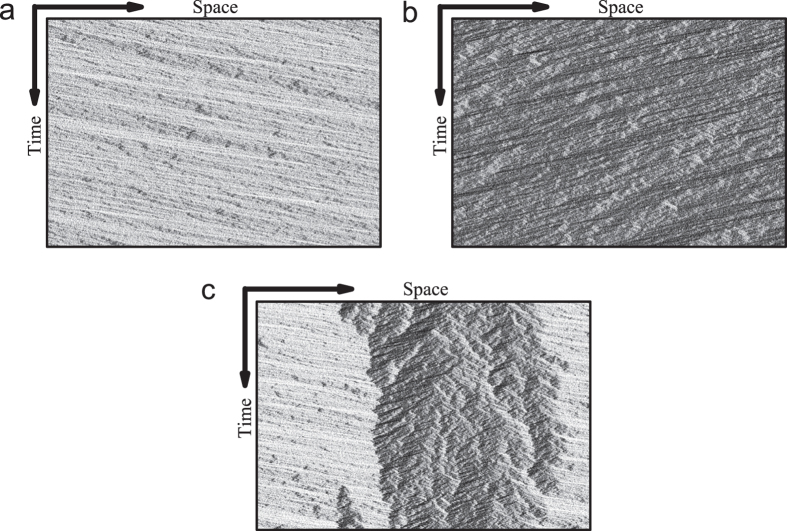
The spatiotemporal patterns in the bidirectional two-lane model. (**a**) Homogeneous state, the density *ρ* = 0.29, the parameter *p* = 0.7. (**b**) Homogeneous state, the density *ρ* = 0.63, the parameter *p* = 0.7. (**c**) Phase separation state, the density *ρ* = 0.4, the parameter *p* = 0.4. Here we show patterns on one of the two lanes, patterns on the other lane are similar.

Figure 4(a) shows the plot of average velocity versus particle density in the bus route model. Figure 4(b) shows the plot of flow rate versus particle density in the bidirectional two-lane model. In the bus route model, when the density is above a critical value *ρ*_*c*_, the system is macroscopic homogeneous, see Fig. 5(a). However, below *ρ*_*c*_, bus bunching occurs and the system becomes macroscopically non-homogenous, see Fig. 5(b). In the bidirectional two-lane model, the system is homogenous when density is below *ρ*_*c*1_ or above *ρ*_*c*2_, see Fig. 6(a,b). When the density is in the range *ρ*_*c*1_ < *ρ* < *ρ*_*c*2_, the system is non-homogeneous because phase separation occurs, see Fig. 6(c).

**Figure 7:**
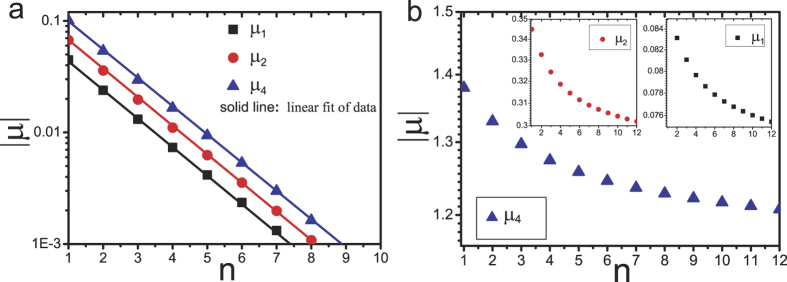
Plot of absolute value of correlation coefficients 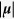 versus distance *n* in the bus route model with (a) *p*  = 0.7, *λ* = 0.2, *ρ* = 0.4 and (b) *p* = 0.5, *λ* = 0.02, *ρ* = 0.2. The symbols denote the simulation results with system size *L* = 6000, the lines indicate the linear fit.

**Figure 8:**
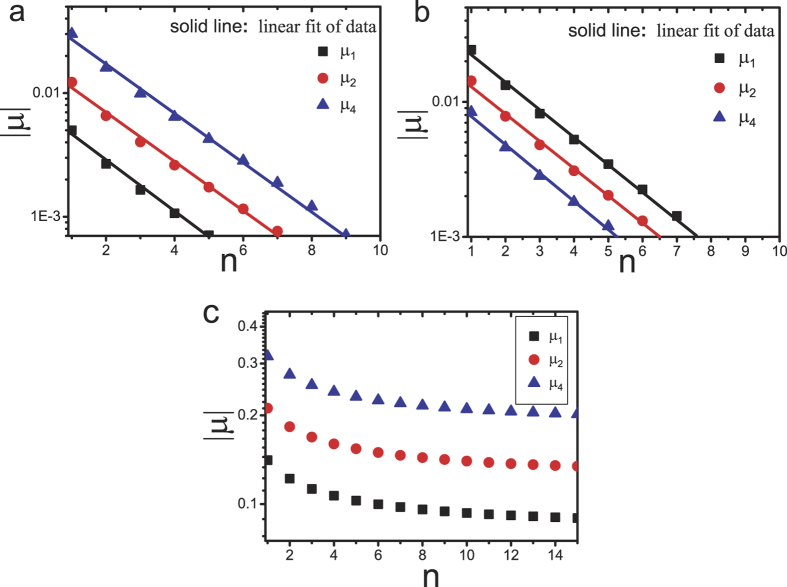
Plot of absolute value of correlation coefficients 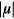 versus distance *n* in the bidirectional two-lane model with (a) *p*  = 0.7 and *ρ* = 0.29, (b) *p* = 0.7 and *ρ* = 0.63, (c) *p* = 0.4 and *ρ* = 0.4. The symbols denote the simulation results with system size *L* = 6000, the lines indicate the linear fit.

Figures 7 and 8 show the correlation in the bus route model and in the bidirectional two-lane model. One can see that when the system is homogenous, the correlation decays exponentially (Figs 7(a) and 8(a,b)). However, when the system is not homogenous, the correlation does not decay exponentially, which bends upward in the semi-log plane (Figs 7(b) and 8(c)).

Our studies thus demonstrate that the exponential decay behavior of correlation might be a universal property in a broad class of 1D driven diffusive systems with macroscopic homogeneous state. This might be because there is a specific correlation length, which should be the same for homogeneous cases. However, such one length does not exist for inhomogeneous cases. Of course further efforts are needed upon this issue in the future work.

## Methods

### Mean Field Analysis

**Figure 9:**
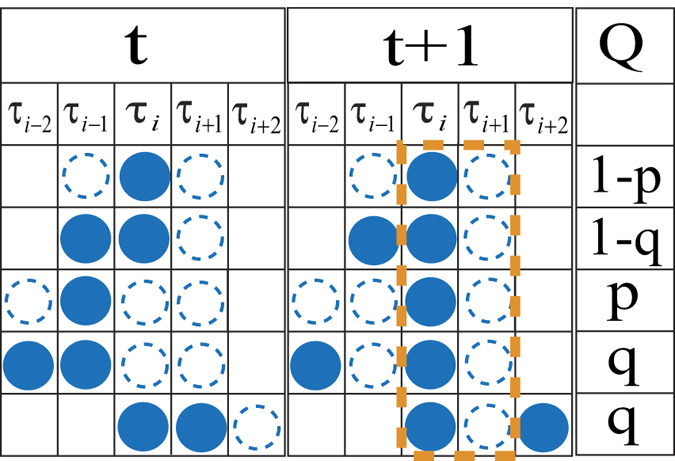
Sketch of possible evolutions of 3 or 4-clusters and corresponding transition probabilities to develop into the situation *τ*_*i*_ = 1, *τ*_*i*+1_ = 0 (the configuration in the dotted box). The circles in the first two columns represent the states of the sites at time *t* and *t* + 1, respectively. The last column presents the corresponding transition probabilities. The dotted-open and filled circles correspond to empty sites and sites occupied by particles, respectively. The absence of circles means whether the site is empty or occupied does not matter.

In the mean field analysis, the two equations


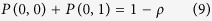



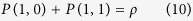


can be written easily. The third equation can be obtained via the master equation for *P*(1, 0) according to the evolution configurations as shown in Fig. 9, which presents the configurations at *t* and *t* + 1 as well as the corresponding transition probabilities. The first column shows all those configurations which can give rise to the configurations shown in the second column. The second column lists exhaustive clusters configurations with *τ*_*i*_ = 1, *τ*_*i*+1_ = 0. The third column presents the corresponding transition probabilities from the configurations in the first column to the corresponding configurations in the second column. Thus,


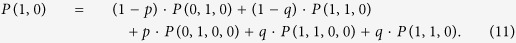


In the 2-cluster mean field analysis, 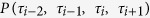 can be expressed mathematically as^13^,^43^,^44^,^45^


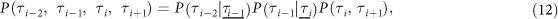


where


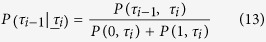


is 2-cluster conditional probability. Similarly, 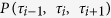 and 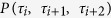 can be expressed as


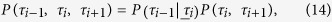



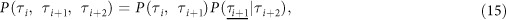


where


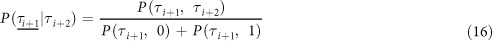


is also 2-cluster conditional probability. So the probabilities of 4-clusters and 3-clusters involved in the right-hand-side of Eq. (11) can be expressed as follows


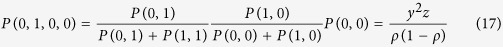



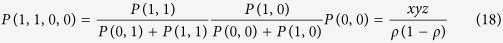



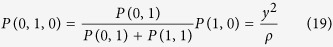


Note that the first *P* (1, 1, 0) in Eq. (11) corresponds to 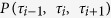, thus


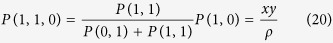


The second *P* (1, 1, 0) in Eq. (11) corresponds to 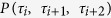, thus


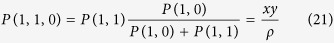


which is identical to Eq. (20). This feature can be easily proved in the general case, since


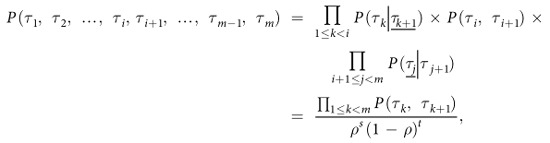


where 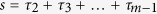 and *t* = *m* − 2 − *s*. This is independent of the location of *i*.

Substituting Eqs (17)–(21) into Eq. (11), we have the third equation about *x*, *y*, *z*


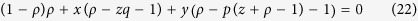


Solving the three Eqs (9), (10) and (22), we can obtain *x*, *y*, *z* as shown in Eqs (1)–(3).

### Correlation Coefficient Analysis

Now we derive the correlation coefficient *μ*_1_. We denote


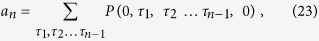


which can be written as


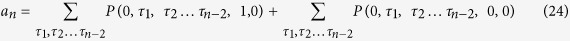


Since


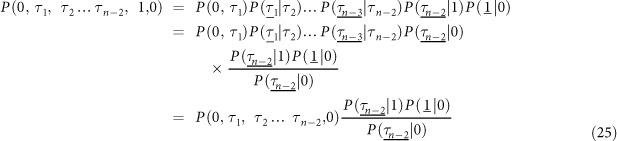


and


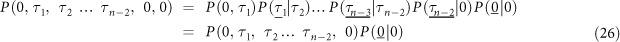


One has


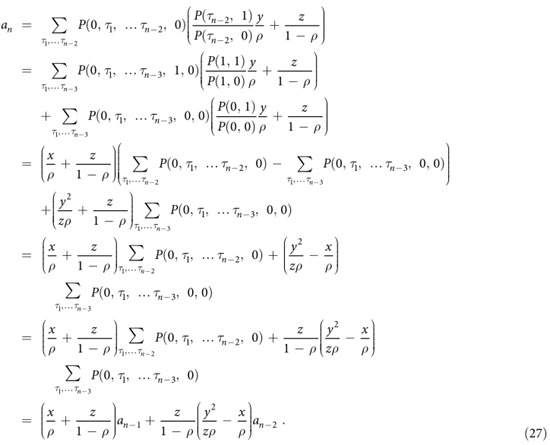


Substituting Eq. (27) into Eq. (5) and simplifying, we can obtain


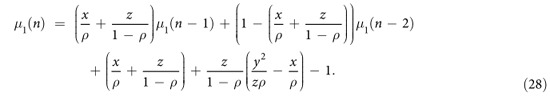


Since *z* = 1 − *ρ* − *y* and *x* = *ρ* − *y*, one can easily prove


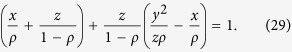


Thus


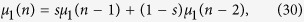


where


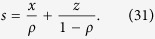


From Eq. (30), we can easily prove that


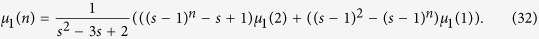


via mathematical induction method. Substituting 
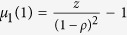
 and 
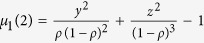
 into Eq. (32), one can derive *μ*_1_(*n*), *μ*_2_(*n*), *μ*_3_(*n*) and *μ*_4_(*n*) as shown in Eqs (6)–(8).

## Additional Information

**How to cite this article**: Hao, Q.-Y. *et al.* Exponential decay of spatial correlation in driven diffusive system: A universal feature of macroscopic homogeneous state. *Sci. Rep.* **6**, 19652; doi: 10.1038/srep19652 (2016).
